# When the Sum of the Parts Tells You More Than the Whole: The Advantage of Using Metagenomics to Characterize *Bartonella* spp. Infections in Norway Rats (*Rattus norvegicus*) and Their Fleas

**DOI:** 10.3389/fvets.2020.584724

**Published:** 2020-10-29

**Authors:** Chelsea G. Himsworth, Kaylee A. Byers, Champika Fernando, Laura Speerin, Michael J. Lee, Janet E. Hill

**Affiliations:** ^1^Canadian Wildlife Health Cooperative—British Columbia, Abbotsford, BC, Canada; ^2^School of Population and Public Health, University of British Columbia, Vancouver, BC, Canada; ^3^Animal Health Center, British Columbia Ministry of Agriculture, Abbotsford, BC, Canada; ^4^Department of Interdisciplinary Studies, University of British Columbia, Vancouver, BC, Canada; ^5^Biodiversity Research Center, University of British Columbia, Vancouver, BC, Canada; ^6^Department of Veterinary Microbiology, University of Saskatchewan, Saskatoon, SK, Canada

**Keywords:** *Bartonella* spp., fleas, metagenomics, rats, *Rattus norvegicus*, *Nosopsyllus fasciatus*, urban

## Abstract

Urban Norway rats (*Rattus norvegicus*) are a reservoir for *Bartonella* spp. - a genus of zoonotic bacteria transmitted by hematophagous vectors, particularly fleas. Rats and fleas may be infected with more than one *Bartonella* species; however, mixed infections may be difficult to detect using culture and/or mono-locus PCR. We set out to characterize *Bartonella* spp. using *gltA* PCR and Sanger sequencing on blood (*n* = 480) and *Nosopsyllus fasciatus* flea pools (*n* = 200) obtained from a population of urban Norways rats from Vancouver, Canada. However, when contamination of a subset of flea pools necessitated the use of a second target (*ssrA*) and the results of *gltA* and *ssrA* were discordant, a metagenomic approach was used to better characterize the *Bartonella* spp. present in these samples and our objective transitioned to comparing data obtained via metagenomics to those from PCR/sequencing. Among the *Bartonella* spp.-positive rats (*n* = 95), 52 (55.3%), and 41 (43.6%) had Sanger sequences consistent with *Bartonella tribocorum* and *Bartonella vinsonii*, respectively. One rat had a mixed infection. All sequences from *Bartonella* spp.-positive flea pools (*n* = 85), were consistent with *B. tribocorum*, and re-analysis of 34 bloods of varying *Bartonella* spp. infection status (based *gltA* PCR and sequencing) using *ssrA* PCR showed that the assay was capable of identifying *B. tribocorum* but not *B. vinsonii*. Metagenomics analysis of a subset of PCR-positive blood samples (*n* = 70) and flea pools (*n* = 24) revealed that both *B. tribocorum* and *B. vinsonii* were circulating widely in the study population with 31/70 (44.3%) rats and 5/24 (2.1%) flea pools infected with both species. *B. vinsonii*, however, made up a smaller relative proportion of the reads for samples with mixed infections, which may be why it was generally not detected by genus-specific PCR and Sanger sequencing. Further analysis of 16S−23S ITS sequences amplified from a subset of samples identified the *B. vinsonii* strain as *B. vinsonii* subsp. *berkhoffii* type II. This demonstrates the value of a metagenomic approach for better characterizing the ecology and health risks associated with this bacterium, particularly given that the less dominant species, *B. vinsonii* is associated with greater pathogenicity in people.

## Introduction

*Bartonella* spp. are a genus of zoonotic, intracellular bacteria that infect erythrocytes and endothelial cells (1). Wild rodents are an important reservoir for *Bartonella* spp. and infections in these species are persistent and asymptomatic (1). In people, however, infection with rodent-associated strains can result in septicemia with a broad range of clinical signs (1, 2). *Bartonella* spp. are transmitted among rodents and from rodents to people by hematophagus arthropods, particularly fleas (3).

At least 22 *Bartonella* spp. have been found in 98 rodent species (1). While certain *Bartonella* spp. have been associated with specific rodent hosts, there are significant overlaps in host range, and *Bartonella* species composition varies markedly and somewhat unpredictably among geographic locations (1). Additionally, it is not uncommon for rodents and their fleas to be infected with multiple *Bartonella* spp. (3).

For those engaged in *Bartonella* spp. surveillance and research, mixed *Bartonella* spp. infections pose a significant diagnostic challenge. Specifically, it can be difficult to detect the presence of multiple *Bartonella* spp. in a single sample using traditional diagnostic techniques, such as culture and/or PCR with Sanger sequencing. *Bartonella* spp. are generally difficult to culture (requiring specialized media/growth conditions and prolonged incubation times), and some species are less cultivable than others (4, 5). Additionally, given that *Bartonella* spp. cannot be clearly or consistently differentiated based on colony morphology (5), mixed infections may not be obvious when observed from the plate. If colonies of one *Bartonella* spp. far outnumber that of another, the less common colonies may not be sampled for genetic identification. Similarly, differential abundance of DNA in a mixed-*Bartonella* spp. infection may make low abundance *Bartonella* spp. difficult to detect using PCR and sequencing performed directly on blood and tissues (4). There are a number of genetic loci that have been used to genotype *Bartonella*, including ITS, *gltA, rpoB, ftsZ, ribC, nuoG, ssrA, pap31*, and *groEL*, and primer sets for these loci vary in their ability to detect different *Bartonella* spp. (4). For this reason, it has been suggested that a single marker cannot reliability capture *Bartonella* spp. diversity in a sample. One alternative is the use of multi-locus approaches, and another is metagenomic methods (4), such as deep sequencing of PCR amplified marker genes, which are more sensitive to intra-host bacterial diversity and the presence of mixed infections (5, 6). Identifying *Bartonella* spp. accurately, as well as the presence of mixed infections, is particularly important from a public health perspective, as different species of *Bartonella* have differing pathogenicity in humans (7).

Norway rats (*Rattus norvegicus*) are a reservoir for *Bartonella* spp. in urban centers. The most common flea vector for *Bartonella* spp. among Norway rats is the Oriental rat flea, *Xenopsylla cheopis* (8). *Bartonella* spp. isolated from urban Norway rats and their fleas include *B. tribocorum, B. elizabethae, B. queenslandensis, B. rochalimae, B. phoceensis, B. rattimassiliensis, B. grahamii*, and related subspecies (8–10). In North America, the majority of *Bartonella* spp. research involving urban Norway rats has occurred in the USA, where *Bartonella* spp. identified include *B. tribocorum, B. elizabethae, B. queenslandensis*, and *B. rochalimae*, of which *B. tribocorum* is the most common (11–15). Human infections with rat-associated *Bartonella* spp. have been limited (2, 16). However, the serological prevalence of exposure in urban populations is much higher (up to 52%), particularly among people who use drugs and/or are street-involved (17, 18). Additionally, it is recognized that rodent-associated zoonotic infections are likely misdiagnosed and underdiagnosed in these groups (18, 19).

Little is known about *Bartonella* spp. in urban Norway rats in Canada. A single Canadian study conducted by our research group demonstrated that urban Norway rats (*Rattus norvegicus*) in Vancouver, Canada carry *Bartonella* spp.. Using culture and genus-specific PCR and Sanger sequencing of the citrate synthase (*gltA*) gene, we found that 25% of rats caught in 2011/2012 were *Bartonella* spp. positive, and all carried *B. tribocorum* with identical *gltA* sequences (20). The original purpose of this study was to use *gltA* PCR and Sanger sequencing to identify *Bartonella* spp. in rats and fleas collected during a follow-up study in the same area during 2016/2017. However, when contamination of a subset of flea pools necessitated the use of a second target (*ssrA*) and the results of *gltA* and *ssrA* were discordant, a metagenomic approach was used to better characterize the *Bartonella* spp. present in these samples and our objective transitioned to comparing data obtained via metagenomics to those from PCR and Sanger sequencing.

## Materials and Methods

### Sample Collection

This study was part of a larger project seeking to determine the effects of lethal pest control (kill trapping) on the disease ecology of urban Norway rats (21). Briefly, rats were collected from 36 city blocks in an underserved neighborhood of Vancouver, British Columbia, Canada, during June 2016–January 2017. The study area was divided into 12 groups of 3 contiguous city blocks, of which 5 groups were assigned to the intervention and 7 groups were controls. In control groups, no lethal-trapping occurred; in intervention groups, lethal-trapping occurred only in the central blocks, and the 2 adjacent blocks were designated as non-lethal flanking blocks to account for potential rat displacement along their contiguous alleyways. Each block was bisected by an alleyway and 10 traps were placed along the length of the alleyway.

Prior to trapping, each block was pre-baited (traps were fixed open with bait) for 1 week to improve capture success. Rats were actively trapped for a total of 6 weeks, with discrete 2-week trapping periods designated as either: before, during, or after the intervention. Before and after the intervention, rats were trapped using Tomahawk live rat traps (Tomahawk Live Traps, Hazelhurst, USA) processed, and released. During processing, rats were marked with a laser-etched ear tag (Kent Scientific, Torrington, USA). During the intervention period, rats caught in intervention blocks were euthanized (intracardiac injection of pentobarbital) while in control sites and flanking blocks, capture-mark-release continued. During active trapping, traps were checked 5 days a week by 0,700 h and were reset by 1,600 h; traps were fixed open and baited on the sixth and seventh day to re-acclimatize rats to traps.

Trapped rats were transferred into an inhalation induction chamber (Kent Scientific, Torrington, USA) attached to an isoflurane vaporizer (Associated Respiratory Veterinary Services, Lacombe, Canada). During anesthetization via 5% isoflurane in oxygen, the majority of fleas vacated the rat and were collected directly from the induction chamber. Rats were then brushed thoroughly to dislodge any remaining fleas into a collection bowl before the rat was transferred to a nose cone to maintain anesthesia. Blood was collected from each rat via the jugular and was stored in heparin coated microtainers (BD, Mississauga, Canada).

Rats were allowed to recover fully before being re-released at their site of capture or they were euthanized, according to the study period and group. Individuals that were recaptured 7 or more days following their previous capture were re-sampled. The University of British Columbia's Animal Care Committee (A14-0265) approved all procedures.

### DNA Extraction

Only samples collected before or after the intervention were included for testing (i.e., samples collected during the intervention were omitted). Rat blood and fleas were stored at −80°C. Prior to DNA extraction, fleas were observed under a compound microscope at 40X magnification and identified to species. A pool of up to 5 fleas per rat was surface-sterilized in 10% bleach, rinsed in nuclease-free water, and then rinsed twice in 100% ethanol to remove bacteria from the external body. Cleaned flea pools were crushed using a sterile scalpel. DNA was extracted using QIAgen DNEasy Blood and Tissue Kit according to the manufacturer's protocol.

Genomic DNA extracts were quantified using a NanoDrop Spectrophotometer ND 2000 (Thermo Fisher Scientific, Canada) and the suitability of rat blood and flea pool DNA extracts for PCR was confirmed using conventional PCR assays targeting host genes cytochrome B (*cyt*B), or cytochrome oxidase subunit 2 (*cox*II) for rats and fleas, respectively.

Specifically, primers JH0792 and JH0793 (Supplementary Table 1) were designed to amplify a 224 bp fragment of *R. norvegicus cyt*B (Genbank accession number KY356141.1). A positive control was created by ligating the 224 bp amplicon from blood sample BA-1 into cloning vector pGEM T-Easy (Promega, USA). All genomic DNA extracts from blood samples were diluted 1:10 prior to use as a template in PCR. Thermocycling parameters were as follows: an initial denaturing step at 95°C for 3 min, and 40 cycles of denaturing, annealing, and extension at 95°C for 15 s, 60°C for 15 s, 72°C for 20 s, respectively, and a final extension at 72°C for 5 min. Amplicons were stored at 4°C if not immediately run in a 1% agarose gel containing ethidium bromide and visualized under UV light. Samples producing the expected 224 bp product were considered suitable for screening with *Bartonella* specific PCR.

For flea pool DNA extracts, PCR with primers JH0784 and JH0785 (Supplementary Table 1) (22) targeting a 550 bp region of flea *cox*II was performed. DNA extracts were run in a conventional PCR with the following steps: initial denaturation at 95°C for 5 min, 40 cycles of denaturation at 94°C for 40 s, annealing at 66.1°C for 1 min, and extension at 72°C for 1 min. After cycling a final extension at 72°C was performed for 7 min and held at 4°C if not immediately run in a 1% agarose gel containing ethidium bromide and visualized as previously described. The 550 bp *cox*II amplicon from *Ceratophyllus vagabundus vagabundus* (kindly provided by Kayla Buhler) was cloned into pGEM T Easy vector as previously described and acted as a positive control. Samples producing the expected 550 bp product were considered suitable for screening with *Bartonella*-specific PCR.

### *Bartonella* Detection by PCR Targeting *gltA* and *ssRA* and 16S-23S ITS, and by Sanger Sequencing

Primers to detect a 380 bp segment of the *Bartonella* spp. citrate synthase gene (*gltA*) from Hornok et al. (23) were used in a SYBR green real-time PCR. A positive control was produced by ligation of the *gltA* PCR product from a sample provided by Prairie Diagnostic Services, Inc. (Saskatoon, Canada), determined to be *Bartonella vinsonii* based on *gltA* sequence similarity to *Bartonella vinsonii* subsp. *berkhoffii* str. Winnie (Genbank accession number CP003124.1) into vector pGEM T Easy. A standard curve was made from a 10-fold dilution series of the plasmid (10^1^-10^7^ plasmid copies/μL) and used to determine the efficiency of the PCR. The 10^2^ and 10^7^ copies/μL dilutions were used as positive controls in subsequent screening of samples. DNA extracted from *C. vagabundus* determined to be *Bartonella* negative were pooled and used as a negative control for both rat and flea samples.

Samples were run in a real-time PCR in duplicate in blocks of 20 along with the two positive controls and two negative controls described and with a no template control. A QIAgility robot (QIAgen, Germany) loaded 8 μL of PCR master-mix containing 1× SYBR green (Bio-Rad, USA) 0.4 μM of each of primers JH0782 and JH0783 (Supplementary Table 1), and 2 μL of DNA template. The PCR was run on a Bio-Rad CFX Connect System with the following parameters: an initial denaturation at 95°C for 3 min, 40 cycles of denaturation, annealing, and extension at 95°C for 10 s, 57°C for 10 s and 72°C for 30 s, respectively. A dissociation curve was run after amplification. Real-time PCR data was analyzed using the end-point analysis protocol in CFX Manager (Bio-Rad, USA). Samples for which both duplicates were positive with the correct melt peak (80°-80.5°C), were considered positive for *Bartonella*.

It was discovered that 60 flea pool DNA extracts had *gltA* PCR-positive extraction controls as a result of contamination (likely with the positive-control plasmid), therefore an alternative assay was implemented to analyze the flea samples. A probe based real-time PCR assay was performed to detect a 302 bp fragment of the *ssrA* gene of *Bartonella* spp. in pooled flea genomic DNA samples according to Diaz et al. (24). pGEM T Easy vector containing the *ssrA* amplicon from a positive sample was used as a positive control and to determine the efficiency of the PCR as described for *gltA*. The 10^7^ and 10^2^ (plasmid copies/μL) standards were included as positive controls in subsequent screening of flea pools. Negative controls were the same as were used in the *gltA* assay. Real-time PCR was performed in duplicate in batches of 20 along with the two positive controls, two negative sample controls, a no template control and an extraction control. A Qiagility robot (Qiagen, Germany) loaded 8 μL of PCR master-mix containing 1× IQ Supermix (Bio-Rad, USA) 0.1 μM of each of primers JH0801 and JH0802, 0.2 μM of probe JH0806 (Supplementary Table 1), and 2 μL of DNA template. PCR was conducted on a Bio-Rad CFX Connect system at 95°C for 2 min followed by 45 cycles of (15 s at 95°C, 1 min at 60°C) with data collection in the FAM channel. Real-time PCR data was analyzed using the end-point analysis protocol in CFX Manager (Bio-Rad, USA). Samples for which both duplicates were called positive were considered positive for *Bartonella*.

Real-time PCR products from *gltA* (*n* = 98) or *ssrA* (*n* = 102) of *Bartonella* were purified using QIAquick PCR Purification kit (Qiagen, Germany), and sequenced with their respective amplification primers (Macrogen, South Korea). Raw sequence reads were processed and assembled using PreGap4 and Gap4 programs in the Staden package (25). Finished amplicon sequences were aligned to the NCBI Genbank non-redundant nucleotide database using BLASTn for identification.

In order to determine whether PCR-negative rat blood samples could be a result of inhibition, 11 randomly selected samples that had been negative on the initial *gltA* PCR were diluted 1:10, 1:100, and 1:1,000 in TE buffer and analyzed by *gltA* PCR in duplicate along with two positive controls, two negative controls, and one no-template control.

Given that the *gltA* target is thought to be more reliable than the *ssrA* for identifying a range of *Bartonella* spp. (4), a randomly selected group of *gltA*-negative rat bloods (*n* = 10), *gltA*-positive bloods that were found to contain *B. tribocorum* on Sanger sequencing (*n* = 11), and *gltA*-positive bloods that were found to contain *B. vinsonii* on Sanger sequencing (*n* = 13) were analyzed using the *ssrA* PCR in duplicate with sequencing of any resulting amplicons.

To determine the genotype of *B. vinsonii* subsp. *berkoffii* we amplified the 16S−23S ITS region using primers 321 and 983 s as described by Maggi et al. (26) (Supplementary Table 1). PCR products were purified and sequenced as described above.

### Metagenomic Sequencing of *gltA* Amplicon of *Bartonella* in Rat Blood and Flea Pools

The contaminated batches of flea pools (*n* = 37) were excluded from the metagenomic analysis. To investigate the occurrence of mixed *Bartonella* spp. infections, remaining rat and flea samples that were positive for *Bartonella* either by *gltA* PCR or *ssrA* PCR were selected for deep sequencing of *gltA* amplicons. The *gltA* primers of Norman et al. (22) (Supplementary Table 1) were modified with the addition of Illumina adapters, resulting in a 487 bp amplicon. Amplicon libraries were constructed according to Illumina 16S metagenomics protocol (Part # 15044223 Rev. B) with modifications in the amplicon generation step, and PCR product purification steps. A 487 bp *gltA* amplicon was generated with 2 μl of genomic DNA in a 50 μl PCR reaction containing 2.5 U of Platinum *Taq* DNA polymerase (Invitrogen), 2.5 mM MgCl_2_, 50 mM KCl, 10 mM Tris/HCl pH 8.3, 250 μM each of dNTPs and 0.1 μM of each of primers *gltA*F and *gltA*R (Supplementary Table 1). Reactions were incubated at 95°C for 3 min followed by 40 cycles of (10 s at 94°C, 10 s at 57°C and 30 s at 72°C) and a final extension of 5 min at 72°C. PCR amplicons were purified with 40 μl of NucleoMag beads (TaKara Bio, USA). Addition of index sequences was performed according to the Illumina protocol and indexed amplicons were purified using the same NucleoMag bead protocol. Libraries were diluted to 8 pM prior to loading on to MiSeq (Illumina, USA), and a 500 cycle (2 × 250, V2 Nano) sequencing run was performed. Three sequencing runs were performed. Extraction negative controls and no template controls were also carried through the library preparation and sequencing.

Amplification primer sequences were removed from raw FASTQ data using cutadapt (27). The resulting reads were trimmed for quality with Trimmomatic (28) using a quality score of 30 and a minimum length of 100. Unique sequence variants were identified using DADA2 (29) within QIIME2 (version qiime2-2019.10) using a truncation length of 150. Paired reads 1 and 2 (R1 and R2) were analyzed separately. The resulting variant sequences and biom files from DADA2 were exported for further analysis. The biom files were converted to feature tables containing read counts for each unique variant detected in each sample. Variant sequences were aligned with watered_BLAST (30) to a custom database created from 135 publicly available *Bartonella* spp. genomes retrieved from the NCBI Genomes database (20 Jan 2020) for identification. Within a sample the number of reads attributed to each *Bartonella* spp. was converted into a relative proportion of the total *Bartonella* spp. reads and a specific *Bartonella* species was only considered to be present if it accounted for ≥ 1% of the reads within a sample.

### Phylogenetic Analysis of *gltA* Sequences

Partial *gltA* gene sequences were aligned with representatives of rodent-associated *Bartonella* spp. using MUSCLE and the alignment was trimmed to 259 positions common to all sequences. A consensus neighbor-joining tree (500 bootstrap replications) was constructed in MEGA X (31).

### Statistical and Spatial Analysis

The location from which each sample was collected and whether that sample contained *B. tribocorum* or *B. vinsonii* on metagenomic analysis was mapped using ArcGIS 10.0 (ESRI, Redlands, USA). This information was imported into SaTScan^TM^ (Boston, USA) for cluster analysis using a purely spatial Bernoulli model and scanning for areas with high rates of *B. tribocorum* and *B. vinsonii* using a circular window with a maximum spatial cluster size of 50% of the population at risk. The analysis was repeated for the samples collected before the intervention, samples collected after the intervention, and for the total dataset (combining samples collected before and after the intervention).

Among the samples that underwent metagenomic analysis, the proportion of reads from deep sequencing belonging to *B. tribocorum* (vs. *B. vinsonii*) mirrored a binomial distribution, with all samples falling below 34% or above 70%. For this reason, the outcome was dichotomized to indicate the predominance of *B. tribocorum* (>70%) vs. *B. vinsonii* (<34%). Logistic regression was used to assess the individual effects of the intervention, season, and sample type (rat blood vs. flea pool) on the dichotomous outcome of *B. tribocorum* vs. *B. vinsonii*. There was not enough data for multivariable or multi-level modeling. The intervention variable was categorized as samples obtained: before the intervention in any block type (*n* = 43); after the intervention in control blocks (*n* = 35); after the intervention in flanking blocks (*n* = 9), and; after the intervention in intervention blocks (*n* = 6). All statistical analyses were conducted using R (R Development Core Team, Vienna, Austria).

## Results

### *Bartonella* Detection by PCR Targeting *glt*a and *ssr*a and by Sanger Sequencing

**Figure 1:**
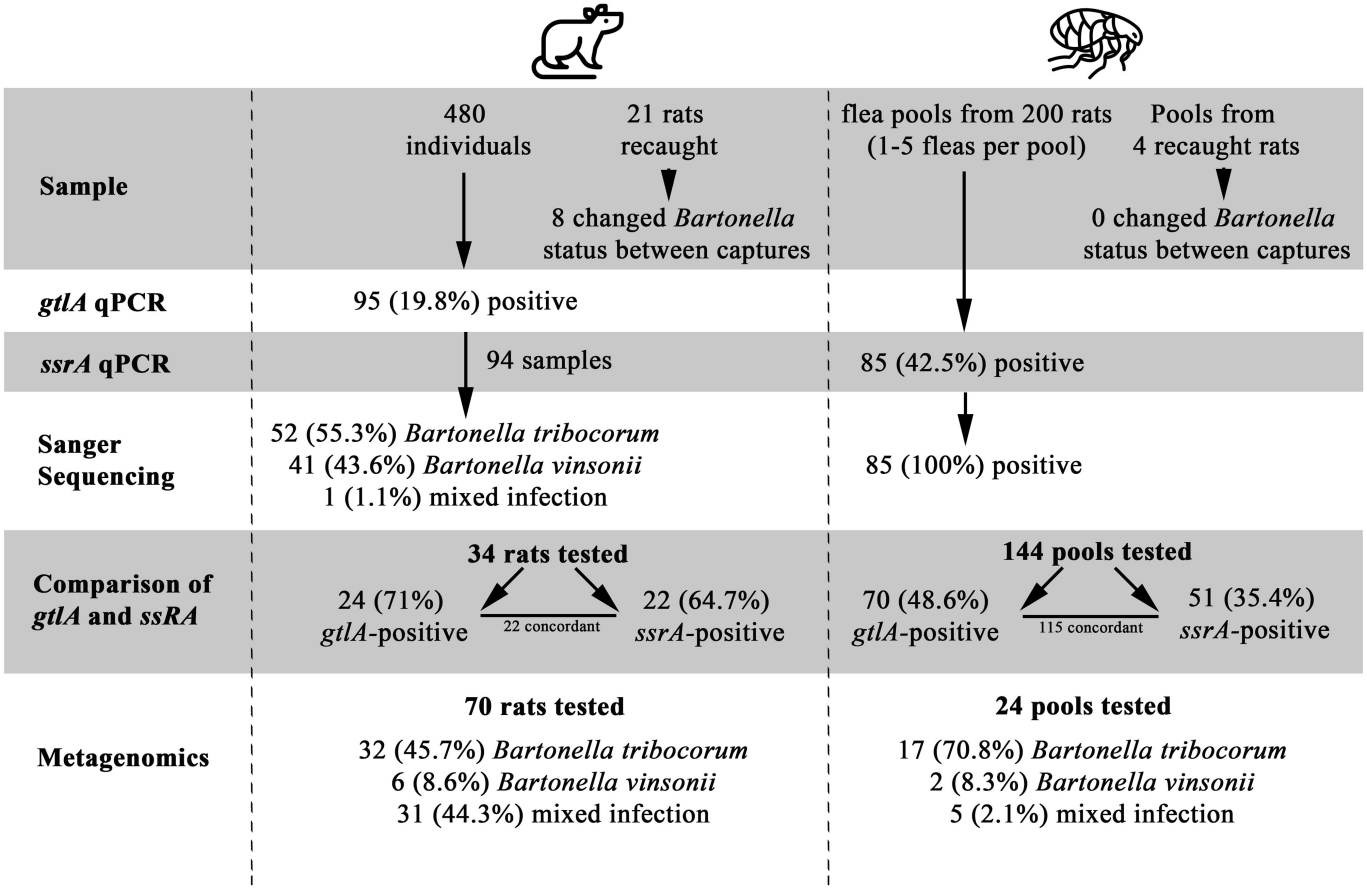
A Flowchart of study results, illustrating the number of samples tested, positivity of samples, and identification of *Bartonella* species. Flea by Grégory Montigny and Lab Rat by HeadsOfBirds from the Noun Project.

A total of 480 rats were included in the study, of which 95 (19.79%) were positive for *Bartonella* spp. based on *gltA* PCR at some point during the study (Figure 1, Supplementary Data Sheet 2). All blood DNA extracts were positive for the rat cytochrome B PCR, and none of the *Bartonella* spp.-negative samples or their dilutions were positive on *gltA* during the inhibition assay, indicating that PCR inhibition was not a factor in the negative results. Sanger sequencing results were available for PCR products from 94 *Bartonella* spp.- positive rats, of which 52 (55.3%) were *B. tribocorum*, 41 (43.6%) were *B. vinsonii*, and 1 was a mixture of *Bartonella* spp. (Supplementary Data Sheet 2). Of the 480 rats included in the study, 459 were captured only once and 21 were captured twice. Of the rats that were recaptured, PCR results did not change in 13 individuals, while 4 animals were PCR-positive on the first capture and PCR-negative on the second capture, and 4 were PCR-negative on the first capture and PCR-positive on the second capture (Figure 1).

All fleas were identified as *Nosopsyllus fasciatus* (32). Flea pools were obtained from 200 unique individual rats of which 85 (42.5%) were positive for *Bartonella* spp. on the *ssrA* PCR and all *Bartonella* spp.-positive flea pools were identified as *B. tribocorum* on sequencing. Four of the 200 rats were captured twice and had fleas present upon capture (yielding an additional 4 flea pools). *Bartonella* spp. status of flea pools among re-captured rats did not change between capture events. *gltA* PCR results could be interpreted for 144 flea pools with clean negative control results, and 70/144 (48.6%) were positive.

### *gltA* and *ssrA* PCR Comparison

Among the 204 flea pools, 144 were tested using both the *ssrA* and the *gltA* PCR (60 flea pools were contaminated and dropped from the study). Among these pools, 70 (48.6%) and 51 (35.4%) were positive on the *gltA* and *ssrA* PCR, respectively. A total of 115 samples yielded concordant results, while 24 were *gltA*-positive and *ssrA*-negative and 5 were *ssrA*-positive and *gltA*-negative.

Among the subset of rat blood samples that were *gltA*-negative (*n* = 10), *B. tribocorum-*positive based on *gltA* sequencing (*n* = 11), and *B. vinsonii*-positive based on *gltA* sequencing (*n* = 13), 0/10, 10/11 and 2/13 were positive on *ssrA* PCR. All *ssrA* amplicons were identified as *B. tribocorum* by sequencing except one, which was a mixture of *Bartonella* spp. (Figure 1).

Because the original validation of the *ssrA* PCR assay included *B. vinsonii* subsp. *vinsonii* and subsp. *arupensis* but not subsp. *berkhoffii*, we aligned the primer and probe sequences to the *ssrA* gene from *B. vinsonii* subsp. *berkhoffii* strain Winnie (Genbank accession NC_020301) to determine if the assay components were compatible with this subspecies. No mismatches were found.

### Metagenomic Sequencing of *gltA* Amplicon of *Bartonella* in Rat Blood and Flea Pools

Sequence data has been deposited to the NCBI Sequence Read Archive and is associated with BioProject PRJNA645176. A total of 94 PCR-positive samples met the criteria of generating at least 1,000 reads and being associated with no-template controls and extraction negative controls yielding <10 reads. A total 375,000 pairs of reads remained following quality filtering (average 4,000 pairs of reads per sample). Two samples were removed from the subsequent analysis because they yielded <250 quality filtered reads. It should be noted that the metagenomic sequence data is not quantitative, so number of reads is not reflective of actual *Bartonella* spp. load in a sample.

The length of the amplicon precluded assembly of R1 and R2, therefore the read data were analyzed separately. DADA2 identified 19 unique R1 sequence variants; 9 were 97–100% identical to *B. tribocorum* and 10 were 97–100% identical to *B. vinsonii* (Supplementary Data Sheet 1). Only 4 of these variants were detected in >3 samples (range 6–93), and these most prevalent variants accounted for 98% of the sequence reads. Results from the R2 analysis were similar and so R1 and R2 counts were combined for further analysis (Supplementary Data Sheet 2). Overall, 49 (52.1%) samples contained only *B. tribocorum*, 8 (8.5%) contained only *B. vinsonii*, and 37 (38.3%) contained both *B. vinsonii* and *B. tribocorum* (32/70 rat blood, 5/24 flea pools).

With regard to the *B. vinsonii*-like sequences, it is of note that there were three R1 variants and two R2 variants that were detected in >1 sample. One of the R1 variants was identical to the positive control sequence and two differed from the positive control and each other by one nucleotide. One of the R2 variants was also identical to the positive control and one differed by a single nucleotide.

Among the samples that contained only *B. tribocorum* on metagenomics (32 rat bloods and 17 flea pools), all had been positive for *B. tribocorum* by PCR and Sanger sequencing except for 4 rat bloods, which were positive for *B. vinsonii* on *gltA* sequencing and one flea pool that was *gltA* positive not sequenced. Among the samples that contained only *B. vinsonii* on metagenomics (6 rat bloods and 2 flea pools) all were positive for *B. vinsonii* by PCR and Sanger sequencing except for the two flea pools, which were *gltA* positive but not sequenced. Among the 37 samples that contained both *B. vinsonii* and *B. tribocorum* on metagenomics (32 rat bloods and 5 flea pools), 22 (18 rat bloods and 4 flea pools) were positive for *B. tribocorum* by PCR and Sanger sequencing, 13 (all rat bloods) were positive for *B. vinsonii* by PCR and Sanger sequencing, and one flea pool was positive on *gltA* PCR but not sequenced. Among the 22 samples that contained both *B. tribocorum* and *B. vinsonii* on metagenomics and *B. tribocorum* on PCR and Sanger sequencing, the median proportion of *B. tribocorum* reads was 91.52 (range = 22.37–98.21). Among the 13 samples that contained both *B. tribocorum* and *B. vinsonii* on metagenomics and *B. vinsonii* on PCR and Sanger sequencing, the median proportion of *B. vinsonii* reads was 91.25 (range = 66.83–97.62).

There were eight instances in which the rat and the flea pool from that rat were both collected at the same time and underwent metagenomic analysis. Among these instances, five rats and their corresponding flea pools yielded concordant results (i.e., the same *Bartonella* spp. composition) and three rats and their corresponding flea pools yielded discordant results. Three rats were re-captured and re-tested and both blood samples underwent metagenomic analysis. The *Bartonella* spp. composition remained the same in two rats and changed from 100% *B. vinsonii* to 98% *B. tribocorum* in one rat, which was not from a block that was subject to the intervention.

### Relationship to Previously Described Rat-Associated *Bartonella* spp.

**Figure 2:**
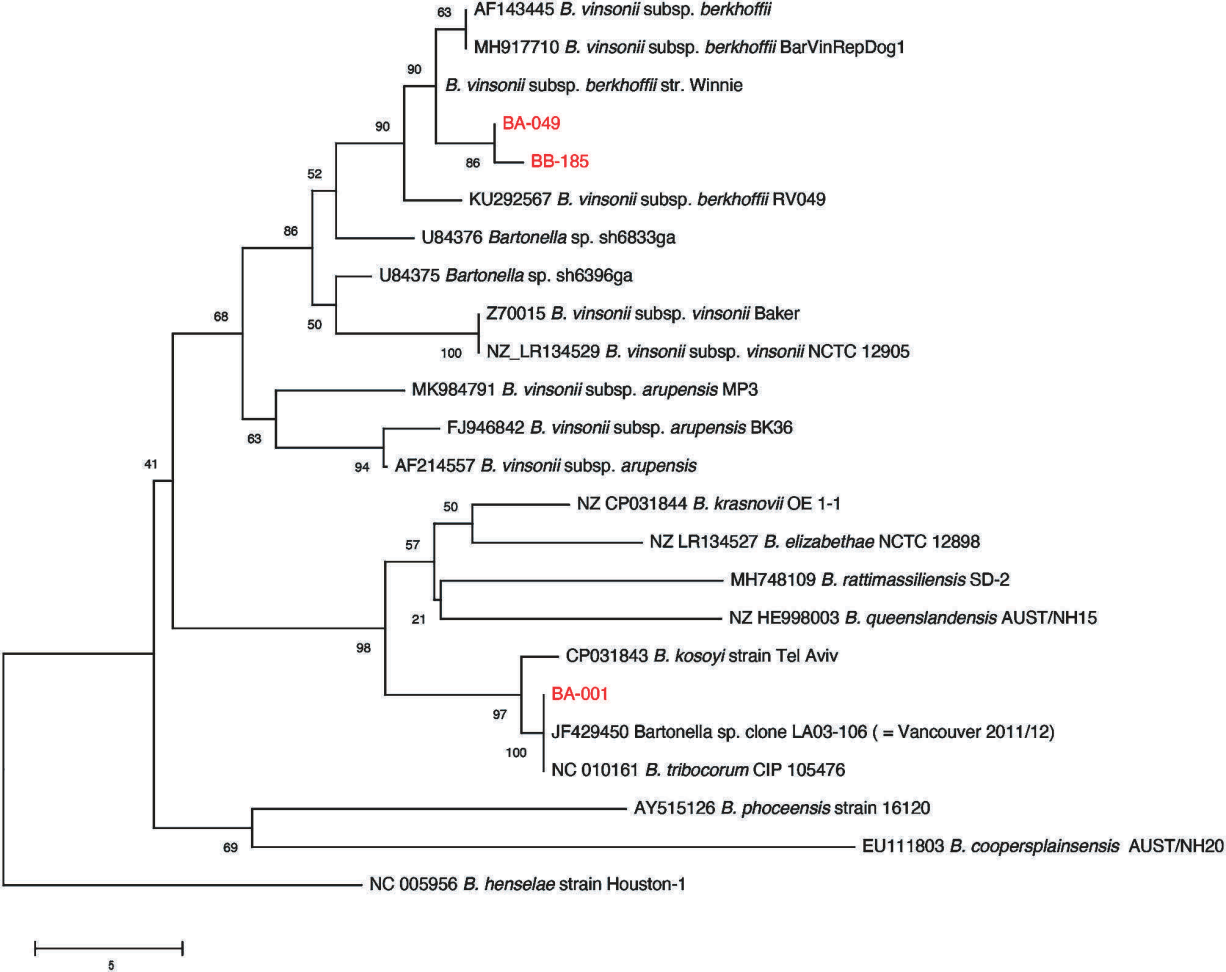
Neighbor-joining tree based on an alignment of partial *gltA* gene sequences. Study sequences were aligned to representatives of rodent-associated *Bartonella* spp. using MUSCLE and the alignment trimmed to 259 positions common to all sequences. The tree is a consensus of 500 bootstrap iterations with the percentage of replicate trees in which the associated taxa clustered together shown at each node. Sequences determined in the current study are shown in red. The *gltA* sequence of an isolate from a previous study of Vancouver rats is indicated. Scale bar indicates number of nucleotide differences.

Multiple sequence alignment of 90 available *glt*A PCR products from the original sample screening resulted in the identification of two distinct *B. vinsonii* like sequences, and one *B. tribocorum* sequence (GenBank accession numbers MT741530, MT741531, MT741532). Phylogenetic analysis of the study sequences and publicly available *gltA* sequences from rodent-associated *Bartonella* spp. indicated that the *B. tribocorum glt*A was identical to an isolate detected in a previous study of rats in Vancouver, Canada (20) (Figure 2). The two *B. vinsonii-*like sequences clustered with subsp. *berkhoffii* with good bootstrap support. It is of note that one *B. vinsonii*-like sequence (BB-185) was identical to the positive control sequence, while the other differs by one nucleotide. That being said, all of the *B. vinsonii* subsp. *berkoffii* sequences included in the tree differ by a maximum of two nucleotides and several of the reference sequences are identical to one another (e.g., AF143445—human clinical isolate from France and MH917710—canine isolate from Mexico).

Maggi et al. (26) proposed a typing scheme for *B. vinsonii* subsp. *berhoffii* based on sequencing of the 16S-23S ITS and *pap*31 loci. Ten samples with high proportions of *B. vinsonii* like reads in the metagenomic study were selected for 16S-23S ITS PCR and sequencing. Three of those samples yielded sufficient PCR product for sequencing, and all of them were identical to the reference sequences for type II (Genbank accession number MT935659).

### Statistical and Spatial Analysis

**Figure 3:**
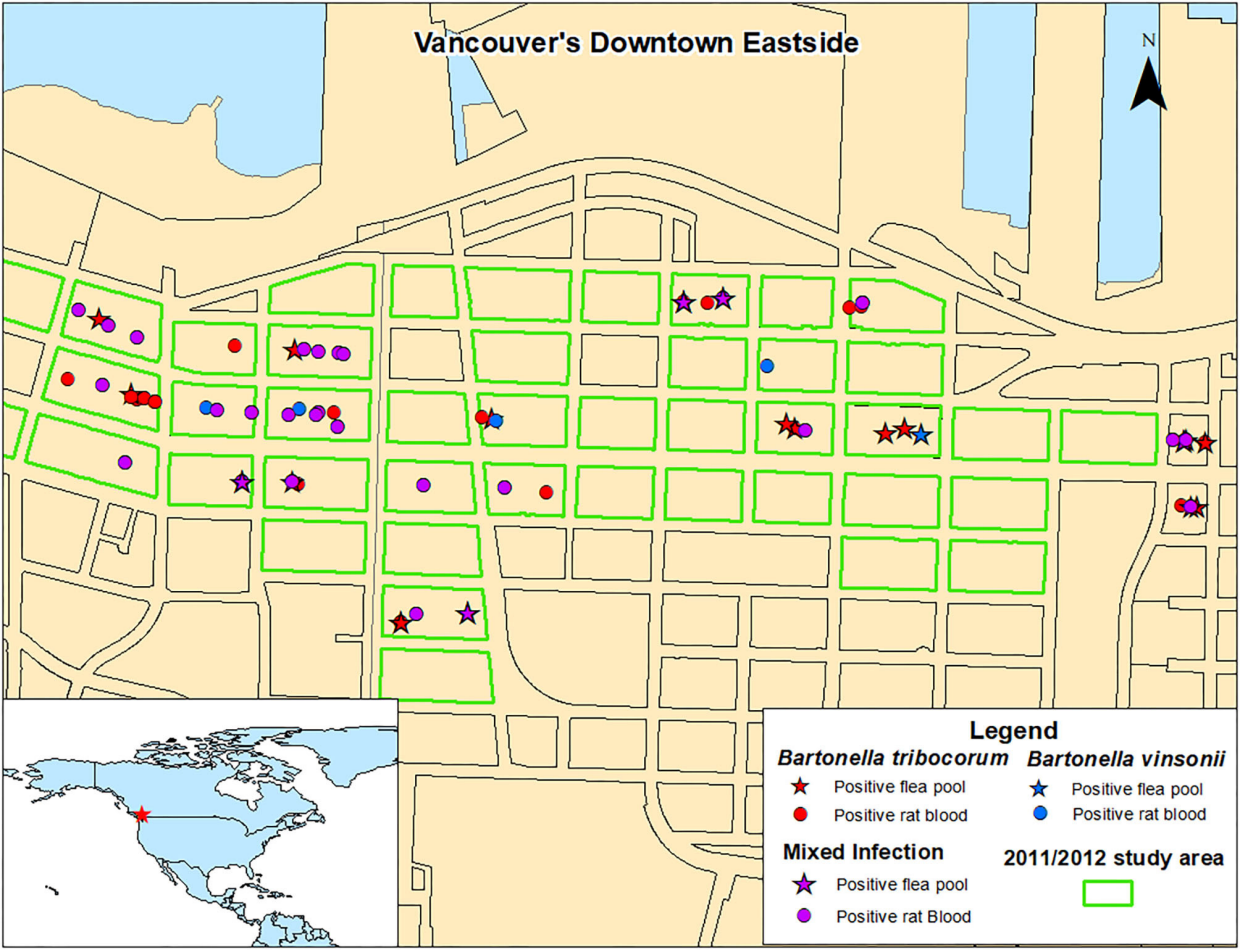
Distribution of Norway rats and rat flea pools carrying *B. tribocorum, B. vinsonii*, or both based on metagenomic analysis.

There were no significant spatial clusters of *B. vinsonii* or *B. triborocum* in any of the datasets considered for investigation (Figure 3). Compared to the period before the intervention, the intervention had no effect on the predominance of *B. tribocorum* vs. *B. vinsonii* (OR = 2.7, 95% CI 0.4–53.8, *p* = 0.39), and similarly, there were no changes in the flanking or control blocks. Additionally, there were no significant differences between rat blood vs. flea pool (OR = 0.35, 95% CI 0.08–1.2, *p* = 0.12). However, *B. tribocorum* had a decreased odds of being the predominant species in the fall vs. the winter (OR = 0.12, 95% CI 0.006–0.7, *p* = 0.045).

## Discussion

This study revealed the presence of two distinct *Bartonella* species within this urban Norway rat population—*B. tribocorum* and *B. vinsonii* subsp. *berkhoffii* type II. *B. tribocorum* is one of the most common *Bartonella* spp. found in urban Norway rats (1), and had been previously identified in rats from the neighborhood currently under study (20). However, *B. vinsonii* does not appear to have been identified in *Rattus* spp. before. Although other subspecies of *B. vinsonii* have been found in non-*Rattus* spp. rodents [such as B. *vinsonii* subsp. *vinsonii* in deer mice, (33)], subsp *berkhoffii* is almost exclusively associated with canids, including domestic dogs and coyotes (17, 34). The detection of this subspecies in our samples may represent a spillover event from dogs to rats, which are sympatric within the study area.

The *ssrA* PCR (24) was capable of amplifying *B. tribocorum* but not *B. vinsonii*, and although *gltA* PCR (22) was capable of amplifying both species, Sanger sequencing indicated the presence of a mixed infection in only one sample. Metagenomics, however, revealed that mixed infections were relatively common, particularly in rat blood samples. Metagenomics also revealed that *B. tribocorum* was the dominant species, with *B. vinsonii* being less common both in terms of number of rats and flea pools infected, and proportion of the *Bartonella* spp. burden for rats and fleas infected with both species.

A 2011/2012 study of rats from the same geographic area detected only *B. tribocorum*. The discrepancy between these two studies is not likely a result of *B. vinsonii* distribution as *B. vinsonii* was found throughout the study area with no significant spatial clustering, and the 2011/2012 and 2016/2017 study areas were almost completely overlapping (Figure 3). Similarly, although the 2011/2012 study included only rat bloods, given that the relative abundance of *B. tribocorum* vs. *B. vinsonii* did not differ between rat bloods and flea pools, the inter-study variability is not likely to be attributable to sample type.

This may suggest that *B. vinsonii* was not present in 2011/2012 but was introduced into the population in the ensuing 5 years. Alternatively, it may be the case that *B. vinsonii* was present in 2011/2012 but not detected using the culture and sequencing approach employed. The difficulty associated with identifying mixed *Bartonella* spp. particularly where those species differ with regard to relative prevalence, has been discussed previously (4). Specifically, the genus *Bartonella* spp. are extremely challenging to culture and certain species and strains may be more cultivable than others (4). Additionally, in mixed bacterial infections, rare species may be less likely to be detected because they may be overgrown by more dominant or more rapidly growing species and/or have a lower probability of being selected for biochemical or genetic characterization (6). Even in cases without differences in growth rates, similar colony morphologies would prevent recognition of mixed cultures by visual inspection of agar plates (5).

In this study, we also found that the molecular techniques vary in their ability to identify different *Bartonella* spp. and mixed infections of *Bartonella* spp.. Specifically, PCR for the *ssrA* gene was not capable of detecting *B. vinsonii*, which was detected on PCR targeting the *gltA* gene. It has been suggested that the *gltA* PCR is less biased toward particular *Bartonella* spp. as compared to other targets, including *ssrA*. Interestingly, it has been shown that *ssrA* PCR is capable of detecting *B. vinsonii* subsp. *vinsonii* and *B. vinsonii* subsp. *arupensis* (35). Additionally, based on available sequence data, the primers should also be capable of amplifying *B. vinsonii* subsp. *berkoffii*—the subspecies with which *B. vinsonii* sequences from this study clustered. This may suggest that these rats and fleas were infected by a subspecies or strain of *B. vinsonii* that could not be captured by the *ssrA* primers. This may not be surprising considering that *B. vinsonii* and its subspecies demonstrate much greater sequence diversity than other *Bartonella* spp. (36). The fact that primers for different loci vary in their ability to detect *Bartonella* spp. supports assertions that single-locus (vs. multi-locus and metagenomic) approaches are not reliable for characterizing *Bartonella* spp. diversity *among* animals in a population (4).

Although the *gltA* PCR amplified both *B. tribocorum* and *B. vinsonii* in this rat population, Sanger sequencing of *gltA* PCR products was generally not capable of detecting mixed *Bartonella* spp. infections. Indeed, only one Sanger sequencing result indicated the presence of more than one *Bartonella* spp. while, for the majority of rats carrying both *B. tribocorum* and *B. vinsonii*, Sanger sequencing results reflected only the dominant *Bartonella* species. This is likely a result of the fact that traditional Sanger sequencing provides a consensus result for the pool of PCR products and signals from low prevalence *Bartonella* spp. may be “drowned out” by higher prevalence species (4)—a problem that would not clearly be resolved using multi-locus approaches. In contrast, the next-generation sequencing approach gives results for *individual* PCR amplicon molecules in each PCR product pool, facilitating the detection of rare species and strains. This suggests that metagenomics may be superior to multi-locus PCR for characterizing *Bartonella* spp. diversity *within* an animal.

It is interesting to note that *Bartonella* spp. detection and characterization may be further complicated by infection dynamics within the rodent host and flea vector. For example, we found that different *Bartonella* spp. may be found in rats and the fleas that infest them, which may be a result of the fact the fleas present on the rat at the time of trapping are unlikely to represent the total population of fleas that rat had been exposed to and vice versa. This is particularly the case for *N. fasciatus* (the Northern rat fleas), which, compared to *X. cheopis* (the Oriental rat flea) spends significantly more time in the host's nest rather than on the host itself (37) and may infest multiple hosts. There is very little known about *N. fasciatus* as a vector for *Bartonella* spp.. Indeed, there appears to have been only one other study of *Bartonella* spp. in the context of this flea species. That study was conducted in Thailand and a novel *Bartonella* spp. was detected in *N. fasciatus* from R*attus surifer* (the red spiny rat) (38). This paucity of information is unexpected as *N. fasciatus* is reported to be on commensal Norway rats in temperate regions (37).

Additionally, infection status may change within an individual rat over time. For example, we identified individual rats that transitioned from being *Bartonella* spp. PCR-negative to -positive and from positive to negative between capture events. Metagenomics analysis also showed that one rat was infected with 100% *B. vinsonii* upon first capture and 98% *B. tribocorum* upon second capture. This may represent an infection/recovery events and/or or it could reflect the fact that, within a host animal, *Bartonella* spp. can reside within a tissue reservoir with only periodic “re-seeding” of the blood stream (3). This means that, during certain periods, *Bartonella* spp. may not be detectable in the blood of an infected host. There was also significant variation in the relative proportion of *B. tribocorum* vs. *B. vinsonii* at a population level among seasons. The prevalence of *Bartonella* spp. within this rat population has been shown to vary by season (20) and these results suggest that seasonal variations in pathogen ecology may also impact *Bartonella* species composition. That being said, it is worth noting that the metagenomic sequence data is not quantitative, so the degree to which changes in *Bartonella* species composition reflects changes in *Bartonella* spp. load cannot be determined. Future studies should consider quantitative comparative metagenomic approaches to facilitate intra- and inter-study comparisons of *Bartonella* spp. ecology (39).

An important limitation of this work is the fact that 60 flea pool DNA extracts were contaminated with the *gltA Bartonella vinsonii* subsp. *berkhoffii* positive-control plasmid—the same *Bartonella* subspecies that was subsequently detected in uncontaminated samples by Sanger sequencing and metagenomics. However, we did take a number of steps to monitor and mitigate the issue of contamination. Specifically, extraction and no-template controls were used rigorously throughout, for the mono-locus PCR analysis, all fleas were re-tested using a separate locus (*ssrA*) and positive control, and contaminated flea samples were excluded from the metagenomic analysis entirely. Additionally, the majority of samples were *Bartonella* spp.-negative, among the *Bartonella* spp.-positive samples, the majority contained only *B. tribocorum*, and *B. vinsonii* was not limited to a particular extraction batch. Finally, there were two *B. vinsonii* subsp *berfhoffii*-like sequences in the samples that were distinct from each other and from the positive control. In combination, this indicates that widespread, undetected contamination was unlikely. Unfortunately, a high level of sequence conservation across the amplified segment of the *B. vinsonii* gene makes it difficult to rule out contamination based on comparing sequences from the samples to that of the positive control. Specifically, there were several *B. vinsonii* variants detected, some of which were identical to the positive control sequence and some of which differed by a single nucleotide. However, many of the reference sequences are identical across this region or differed by single nucleotides. That being said, the fact that we were able to amplify non-identical variants independently from multiple samples further decreases the likelihood that our findings are a result of contamination. Finally, the 16S−23S ITS sequence of *B. vinsonii* subsp. *berkhoffii* type II—a target not included in the positive control plasmids—was detected by PCR and sequencing in several samples.

The ability to detect mixed *Bartonella* spp. infections in rats is important from a public health perspective for two reasons. Firstly, *Bartonella* spp. differ with regard to their pathogenicity for humans. While both *B. tribocorum* and *B. vinsonii* have been associated with human illness (2), in this study, the less dominant species, *B. vinsonii* subsp. *berkhoffii* type II, appears to pose a greater human health risk than the more dominant one, *B. tribocorum* (7)—a risk that would have been underestimated using traditional *Bartonella* spp. diagnostic methods. Secondly, an understanding of the full complement of zoonotic *Bartonella* spp. strains circulating in urban rats is important in order to accurately assess human exposure in the absence of active disease. For example, subsequent to the 2011/2012 study, we found evidence of human exposure to *B. tribocorum* in a serosurvey of residents of the study area (40). This study utilized *B. tribocorum* isolated from rats in the 2011/2012 study to produce an indirect immunofluorescence assay. The assay was shown to have minimal cross reactivity with *B. henselae* and *B. quintana* (20) and therefore may not have detected exposure to *B. vinsonii*.

Overall this study highlights the challenges associated with developing an accurate understanding of *Bartonella* spp. in rat populations and supports the recommendations put forward by Kosoy et al. (4) that researchers and diagnosticians utilize multiple PCR targets and/or a metagenomic approach in order to ensure that all *Bartonella* spp. present are identified.

## Data Availability Statement

Sequence data has been deposited to the NCBI Sequence Read Archive and is associated with BioProject PRJNA645176.

## Ethics Statement

The animal study was reviewed and approved by The University of British Columbia's Animal Care Committee (A14-0265).

## Author Contributions

JH, CH, and KB designed the study and prepared the manuscript. KB, ML, and CH conducted the fieldwork and sample collection. CF and LS conducted the lab work. JH performed the bioinformatics analysis. ML and CH performed the statistical and spatial analysis. All authors contributed to the article and approved the submitted version.

## Conflict of Interest

The authors declare that the research was conducted in the absence of any commercial or financial relationships that could be construed as a potential conflict of interest.

## Supplementary Material

Supplementary Table 1 PCR primers and probes used in this study.

Supplementary Data Sheet 1 Unique sequence variants detected in metagenomic study.

Supplementary Data Sheet 2 PCR, Sanger sequencing and metagenomic results.
