# Supplementary material for: When the Sum of the Parts Tells You More Than the Whole: The Advantage of Using Metagenomics to Characterize Bartonella spp. Infections in Norway Rats (Rattus norvegicus) and Their Fleas
Source: Front Vet Sci. 2020 Oct 29;7:584724. doi: 10.3389/fvets.2020.584724 (PMC7658385; doi:10.3389/fvets.2020.584724)
Supplement: Supplementary Table 1 — PCR primers and probes used in this study. [file Table_1.DOCX]

**Table S1.** PCR primers and probes used in this study

| Primer | Sequence (5’- 3’) | Species | Target | Amplicon Size (bp) | Ref. |
| --- | --- | --- | --- | --- | --- |
| JH0782 | GGGACCAGCTCATGGTGG | *Bartonella* | *gltA* | 380 | 23 |
| JH0783 | AATGCAAAAAGAACAGTAAACA | *Bartonella* | *gltA* | 380 | 23 |
| JH0784 | TCTAATATGGCAGATTAGTGC | Flea | *coxII* | 780 | 22 |
| JH0785 | GAGACCAGTACTTGCTTTCAGTCATC | Flea | *coxII* | 780 | 22 |
| JH0792 | CGACGCAGACAAAATCCCAT | Rat | *cytB* | 224 | This study |
| JH0793 | GTTTGTTGGGAATGGAGCGT | Rat | *cytB* | 224 | This study |
| JH0801 | GCTATGGTAATAAATGGACAATGAAATAA | *Bartonella* | *ssrA* | 301 | 24 |
| JH0802 | GCTTCTGTTGCCAGGTG | *Bartonella* | *ssrA* | 301 | 24 |
| JH0806 | FAM-ACCCCGCTTAAACCTGCGACG-BHQ1 | *Bartonella* | *ssrA* |  | 24 |
| *gltA*F | TCGTCGGCAGCGTCAGATGTGTATAAGAGACAGGGGACCAGCTCATGGTGG | *Bartonella* | *gltA* | 487 | This study |
| *gltA*R | GTCTCGTGGGCTCGGAGATGTGTATAAGAGACAGAATGCAAAAAGAACAGTAAACA | *Bartonella* | *gltA* | 487 | This study |
| 321s | AGATGATGATCCCAAGCCTTCTGG | *Bartonella* | ITS | 670 | 25 |
| 983as | TGTTCTYACAACAATGATGATG | *Bartonella* | ITS | 670 | 25 |
