# Supplementary material for: When the Sum of the Parts Tells You More Than the Whole: The Advantage of Using Metagenomics to Characterize Bartonella spp. Infections in Norway Rats (Rattus norvegicus) and Their Fleas
Source: Front Vet Sci. 2020 Oct 29;7:584724. doi: 10.3389/fvets.2020.584724 (PMC7658385; doi:10.3389/fvets.2020.584724)
Supplement: Supplementary Data Sheet 1 — Unique sequence variants detected in metagenomic study. [file Data_Sheet_1.DOCX]

>88da79b12d9b7638f478efb77490f8

AGCCAATGAAGCATGCCTAAAGATGCTACTAGAAATAGGTTCAGTTAAGAGAATTCCTGAATTTATTGCACGTGCAAAAGATAAAAATGATCCTTTCCGTCTTATGGGATTTGGTCATCGAGTCTATAAAAATTATGATCCACGCGCAAA

>48e71516ad91317c276bf75b8690ad

AGCCAATGAAGCATGCCTAAAGATGCTACTAGAAATAGGTTCAGTTAAGAGAATTCCTGAATTTATTGCACGTGCAAGAGATAAAGATGATCCTTTCTGTCTTATGGGATTTGGTCATCGAGTCTATAAAAATTATGATCCACGTGCAAA

>a32ca3c8bda01195383672def7247a

AGCCAATGAAGCATGCCTAAAGATGCTACTAGAAATAGGTTCAGTTAAGAGAATTCCTGAATTTATTGCACGTGCAAAAGATAAAAATGATCCTTTCCGTCTTATGGGATTTGGTCATCGAGTTTATAAAAATTATGATCCACGTGCAAA

>5489c5eefaf06a203c2fa1c775eebe

AGCCAATGAAGCATGCCTAAAGATGCTACTAGAAATAGGTTCAGTTAAGAGAATTCCTGAATTTATTGCACGTGCAAAAGATAAAAATGATCCTTTCCGTCTTATGGGATTTGGTCATCGAGTCTATAAAAATTATGATCCACGTGCAAA

>0b7f0e34898247684a9827d6a03471

AGCCAATGAAGCATGCCTAAAGATGCTACTAGAAATAGGTTCAGTTAAGAGAATTCCTGAATTTATTGCACGTGCAAAAGATAAAAATGATCCTTTCCGTCTTATGGGATTTAGTCATCGAGTCTATAAAAATTATGATCCACGTGCAAA

>9b4f8600549299ba67f75227ec3c9e

AGCCAATGAAGCATGCCTAAAGATGCTACTAGAAATAGGTTAAGTTAAGAGAATTCCTGAATTTATTGCACGTGCAAAAGATAAAAATGATCCTTTCCGTCTTATGGGATTTGGTCATCGAGTCTATAAAAATTATGATCCACGTGCAAA

>e50ab2548f9ec2e63689084b0669e0

AGCCAATGAAGCATGCCTAAAGATGCTACTAGAAATAGGTTCTGTTAAGAGAATTCCTGAATTTATTGCACGTGCAAAAGATAAAAATGATCCTTTCCGTCTTATGGGATTTGGTCATCGAGTCTATAAAAATTATGATCCACGTGCAAA

>302856ef7f471f53c5ef7f9726ae1f

AGCCAATGAAGCATGCCTAAAGATGCTACTAGAAATAGGTTCTGTTAAGAGAATTCCTGAATTTATTGCACGTGCAAAAGATAAAAATGATCCTTTCCTTCTTATGGGATTTGGTCATCGAGTCTATAAAAATTATGATCCACGTGCAAA

>4348268acd6a16df5e4a4e7fce8b8f

AGCCAATGAAGCATGCCTAAAGATGCTACTAGAAATAGGTTCTGTTAAGAGAATTCCTGAATTTATTGCGCGTGCAAAAGATAAAAATGATCCTTTCCGTCTTATGGGATTTGGTCATCGAGTCTATAAAAATTATGATCCACGTGCAAA

>19706aeabb6fe37fae886e134085f6

TGCCAATGAAGCATGCCTAAAGATGCTACTAGAAATAGGTTCAGTTAAGAGAATTCCTGAATTTATTGCACGTGCAAAAGATAAAAATGATCCTTTCCGTCTTATGGGATTTGGTCATCGAGTCTATAAAAATTATGATCCACGTGCAAA

>e2da3ea151e42a91c234e6478839d3

TGCCAATGAAGCATGTCTAAAAATGCTACAAGAAATAGGTTCTATTAAAAGAATTCCTGAATTTATTGCACGTGCAAAAGATAAAAATGATCCTTTCCGCCTTATGGGCTTTGGTCACCGTGTCTATAAAAATTATGACCCACGTGCAAA

>0e7a83f35334a20a6c6ecc8d28ee2e

TGCCAATGAAGCATGTCTAAAAATGCTACAAGAAATAGGTTCTATTAAAGGAATTCCTGAATTTATTGCACGTGCAAAAGATAAAAATGATCCTTTCCGCCTTATGGGCTTTGGTCACCGTGTCTATAAAAATTATGACCCACGTGCAAA

>12b8ce200ec124360e6a698d64cd2b

TGCCAATGAAGCATGTCTAAAAATGCTACAAAAAATAGGTTCTATTAAAAGAATTCCTGAATTTATTGCACGTGCAAAAGATAAAAATGATCCTTTCCGCCTTATGGGCTTTGGTCACCGTGTCTATAAAAATTATGACCCACGTGCAAA

>a1beb4f3331f16d5cfaf83e0ce9631

TGCCAATGAAGCCTTTCTAAAAATGCTACAAGAAATAGGTTCTATTAAAAGAATTCCTGAATTTATTGCACGTGCAAAAGATAAAAATGATCCTTTCCGCCTTATGGGCTTTGGTCACCGTGTCTATAAAAATTATGACCCACGTGCAAA

>b94c58440a0bcf9c133cbb0d19e910

TGCCAATGAAGCATGTCTAAAAATGCTACAAGACATCTTTTCTATTAAAAGAATTCCTGAATTTATTGCACGTGCAAAAGATAAAAATGATCCTTTCCGCCTTATGGGCTTTGGTCACCGTGTCTATAAAAATTATGACCCACGTGCAAA

>6216aee003ac513b9bad7d08594b6d

TGCCAATGAAGCATGTCTAAAAATGCTACAAGAAATAGGTTCTATTAAAAGAATTCCTGAATTTATTGCACGTGCAAAAGATAAAAATGATCCTTTCCGCCTTATGGGCTTTGGTCACTGTGTCTATAAAAATTATGACCCACGTGCAAA

>9de79056970fb016983be714e22b4a

TGCCAATGGAGCATGTCTAAAAATGCTACAAGAAATAGGTTCTATTAAAAGAATTCCTGAATTTATTGCACGTGCAAAAGATAAAAATGATCCTTTCCGCCTTATGGGCTTTGGTCACCGTGTCTATAAAAATTATGACCCACGTGCAAA

>cea096a395e7fc30081173e215d776

TGTCAATGAAGCATGTCTAAAAATGCTACAAGAAATAGGTTCTATTAAAAGAATTCCTGAATTTATTGCACGTGCAAAAGATAAAAATGATCCTTTCCGCCTTATGGGCTTTGGTCACCGTGTCTATAAAAATTATGACCCACGTGCAAA

>f16cb055ecb5a49c2e8f7ff8ee1e43

TGCCAATGAGGCATGTCTAAAAATGCTACAAGAAATAGGTTCTATTAAAAGAATTCCTGAATTTATTGCACGTGCAAAAGATAAAAATGATCCTTTCCGCCTTATGGGCTTTGGTCACCGTGTCTATAAAAATTATGACCCACGTGCAAA

>c6be71c1facbb95a477f2eeea204b0ac

TTTCGGTTGGAAAGCCTAGAGCTTTTAACGTAATACCGGAGTAGAAATCAACATTGGGATAAAGCTTTTTCTCAATAAAATATTCATCATTTAGCGCAATTTTTTCAAGTTCTATCGCAATATCAAGAAGTGGATCATCTTGAATGTTGA

>aee0dd3068863a774f26e368f4f0bf71

TTTCGGTTGGAAATCCTAAAGCTTTTAACGTAATGCCAGAATAGAAATCGACGTTCGGATAAAGCTTTTTTTCAACAAAATATTCATCATTTAAGGCGATTTTTTCCAGTTCTATAGCGATATCAAGAAGTGGATCATTTTGAATGTTGA

>c37cb971b8ea7d86231c01824a95a4f3

TTTCGGTTGGAAATCCTAAAGCTTTTAACGTAATGCCAGAATAGAAATCGACATTCGGATAAAGCTTTTTTTCAACAAAATATTCATCATTTAAGGCGATTTTTTCCAGTTCTATAGCGATATCAAGAAGTGGATCATTTTGAATGTTGA

>620b0c175af01c89011174f061f919dd

TTTCAGTTGGAAAGCCTAGAGCTTTTAACGTAATACCGGAATAGAAATCAACATTGGGATAAAGCTTTTTCTCAATAAAATATTCATCATTTAGCGCAATTTTTTCAAGTTCTATCGCAATATCAAGAAGTGGATCATCTTGAATGTTGA

>c8c3b47c381a6151a803ad368bdd3b7a

CTTCGGTTGGAAATCCTAAAGCTTTTAACGTAATGCCAGAATAGAAATCGACGTTCGGATAAAGCTTTTTTTCAACAAAATATTCATCATTTAAGGCGATTTTTTCCAGTTCTATAGCGATATCAAGAAGTGGATCATTTTGAATGTTGA

>d61586675ddd925f3aab2d54a12f5465

TTTCAGTTGGAAAGCCTAGAGCTTTTAACGTAATACAGGAATAGAAATCAACATTGGGATAAAGCTTTTTCTCAATAAAATATTCATCATTTAGCGCAATTTTTTCAAGTTCTATCGCAATATCAAGAAGTGGATCATCTTGAATGTTGA

>29ee7954158d4aac2daab686f5495800

TTTCGGTTGGAAATCCTAAAGCTTTTAACGTAATGCCAGAATAGAAATCGACATTCGGATAAAGCTTTTTTTCAACAAAATATCCATCATTTAAGGCGATCTTTTCCAGTTCTATAGCGATATCAAGAAGTGGATCATTTTGAATGTTGA

>3df9d3481870b5b684f4fc9289f10d1d

TTTCGGTTGGAAATCCTAAAGCTTTTAACGTAATGCCAGAATAGAAATCGACGTTCGGATAAAGCTTTTTTTCAACAAAATATTCATCATTTAAGGCGATTTTTTCCAGTTCTATAGCGATATCAAGAAGTTGATCATTTTGAATGTTGA

>6e02019ab6c9fd05ac57cf6aaf165b30

TTTCGGTTGGAAATCCTAAAGCTTTTAACGTAATGCCAGAATAGAAATCGACGTTCGGATAAAGCTTTTTTTCAACAAAATATTAATCATTTAAGGCGATTTTTTCCAGTTCTATAGCGATATCAAGAAGTGGATCATTTTGAATGTTGA

>8ed9cae602237d196a3dcde3eaa0c510

TTTCGGTTGGAAATCCTAAAGCTTTTAACGTAATGCCAGAATAGAAATCGACGTTCGGATAAAGCTTTTTTTCAACAAAATATTCATCATTTAAGGCGATTTCTTCCAGTTCTATAGCGATATCAAGAAGTGGATCATTTTGAATGTTGA

>7b2e94a45157b2a567ebdb7488e3d126

TTCCGGTTGGAAAGCCTAGAGCTTTTAACGTAATACCGGAGTAGAAATCAACATTGGGATAAAGCTTTTTCTCAATAAAATATTCATCATTTAGCGCAATTTTTTCAAGTTCTATCGCAATATCAAGAAGTGGATCATCTTGAATGTTGA

>7ffa5927294d179502f52c2942433a9c

TTTCGGTTGGAAAGCCTAGAGCTTTTAACGTAATACCGGAGTAGAAATCAACATTGGGATAAAGCTTTTTCTCAATAAAATATTCATCATTTAGCGCAATTTTTTCAAGTTCTATCGCAATATCAAGAAGTAGATCATCTTGAATGTTGA

>99962ad2012ddc07fd65fad5976b83dc

TTTCGGCTGGAAAGCCTAGAGCTTTTAACGTAATACCGGAGTAGAAATCAACATTGGGATAAAGCTTTTTCTCAATAAAATATTCATCATTTAGCGCAATTTTTTCAAGTTCTATCGCAATATCAAGAAGTGGATCATCTTGAATGTTGA

>bb8b87da94522f9ce4802a3c269d5400

TTTCGGTTGGAAAGCCTAGGGCTTTTAACGTAATACCGGAGTAGAAATCAACATTGGGATAAAGCTTTTTCTCAATAAAATATTCATCATTTAGCGCAATTTTTTCAAGTTCTATCGCAATATCAAGAAGTGGATCATCTTGAATGTTGA

>ee7d242ed76ec9ae798dc06c9a8c2a5d

TTTCGGTTGGAAAGCCTAGAGCTTTTAACGTAATACCGGAGTAGAAATCAACATTGGGATAAAGCTTTTTCTCAATAAAATATTCATCATTTAGCGCAATTTTTTCAAGTTCCATCGCAATATCAAGAAGTGGATCATCTTGAATGTTGA
